# A Functional Signature Ontology (FUSION) screen detects an AMPK inhibitor with selective toxicity toward human colon tumor cells

**DOI:** 10.1038/s41598-018-22090-6

**Published:** 2018-02-28

**Authors:** Binita Das, Beth K. Neilsen, Kurt W. Fisher, Drew Gehring, Youcai Hu, Deanna J. Volle, Hyun Seok Kim, Jamie L. McCall, David L. Kelly, John B. MacMillan, Michael A. White, Robert E. Lewis

**Affiliations:** 1 Eppley Institute, Fred & Pamela Buffett Cancer Center, University of Nebraska Medical Center, Omaha, NE 68198 USA; 2 Department of Biochemistry, UT Southwestern Medical Center, Dallas, TX 75390 USA; 3 Department of Cell Biology, UT Southwestern Medical Center, Dallas, TX 75390 USA; 4 Present Address: Department of Pharmacology, Duke University School of Medicine, Durham, NC 27710 USA; 5 Present Address: State Key Laboratory of Bioactive Substance and Function of Natural Medicines, Institute of Materia Medica, Chinese Academy of Medical Sciences and Peeking Union Medical College, 1 Xian Nong Tan Street, Beijing, China; 6 Present Address: Department of Pathology and Microbiology, University of Nebraska Medical Center, Omaha, NE 68198 USA; 7 Present Address: Severance Biomedical Science Institute, Yonsei University College of Medicine, Avison Biomedical Research Center, 50-1 Yonsei-ro, Seodaemun-gu, Seoul, 120-752 Korea; 8 Present Address: Department of Microbiology, Immunology, and Cell Biology, West Virginia University, Morgantown, WV 26506 USA

## Abstract

AMPK is a serine threonine kinase composed of a heterotrimer of a catalytic, kinase-containing α and regulatory β and γ subunits. Here we show that individual AMPK subunit expression and requirement for survival varies across colon cancer cell lines. While AMPKα1 expression is relatively consistent across colon cancer cell lines, AMPKα1 depletion does not induce cell death. Conversely, AMPKα2 is expressed at variable levels in colon cancer cells. In high expressing SW480 and moderate expressing HCT116 colon cancer cells, siRNA-mediated depletion induces cell death. These data suggest that AMPK kinase inhibition may be a useful component of future therapeutic strategies. We used Functional Signature Ontology (FUSION) to screen a natural product library to identify compounds that were inhibitors of AMPK to test its potential for detecting small molecules with preferential toxicity toward human colon tumor cells. FUSION identified 5′-hydroxy-staurosporine, which competitively inhibits AMPK. Human colon cancer cell lines are notably more sensitive to 5′-hydroxy-staurosporine than are non-transformed human colon epithelial cells. This study serves as proof-of-concept for unbiased FUSION-based detection of small molecule inhibitors of therapeutic targets and highlights its potential to identify novel compounds for cancer therapy development.

## Introduction

The Ras oncogene is activated in more than 40% of colon tumors^1^ and 25%-30% of human cancers overall^2,3^. Despite substantial efforts to develop therapeutics targeting this pathway^4,5^, significant challenges still exist. We demonstrated previously that Kinase Suppressor of Ras 1 (KSR1), a molecular scaffold for the Raf/MEK/ERK kinase cascade, is required to maintain the transformed phenotype of Ras-driven tumor cell lines, but is dispensable for the survival and proliferation of non-transformed cells^6^. Using KSR1 as a reference standard in a RNAi-based gene expression high-throughput screen termed Functional Signature Ontology (FUSION)^7^, we identified and validated the γ1 subunit of AMP-activated protein kinase (AMPK) as a contributor to the survival of human colon tumor cells^6^.

AMPK belongs to a family of serine/threonine kinases highly conserved from yeast to human^8^. AMPK functions as a heterotrimeric complex consisting of a catalytic α and regulatory β and γ subunits^9^. Mammalian AMPK acts as an energy sensing kinase that is activated by an increasing AMP/ATP ratio and by metabolic alterations, such as hypoxia, glucose deprivation, decreased ATP production, or increased energy consumption. AMPK is a substrate for kinases such as LKB1 and CAMKK2, which modulate its activity by phosphorylation of the activation loop on both alpha subunits at threonine 172. During severe stress, AMP binding to the γ subunit allosterically activates AMPK, promoting phosphorylation of the α subunit at threonine 172, and protects it from dephosphorylation^10^.

The role of AMPK in cancer is controversial and has been shown to both support and inhibit tumor growth^6,9,11–21^. Retrospective population-based studies suggest that AMPK may act as a tumor suppressor because metformin, an inhibitor of mitochondrial electron transport complex 1 and an indirect AMPK activator, appears to decrease the risk for cancer^22,23^. While the mechanism through which metformin lowers cancer risk is not fully understood, numerous studies demonstrate the value of metformin as an anti-cancer agent *in vitro*, in preclinical *in vivo* models, and in patients^13,14,19,22,23^. However, the link implicating AMPK as a contributor to the metformin-induced anti-cancer effect is controversial.

One recent study demonstrated that some cancer cells have upregulated cancer-specific ubiquitin ligases (MAGE-A3/6) that promote the degradation of AMPK to allow for increased mTORC1 signaling^20^. Peutz-Jeghers Syndrome, which is characterized by the formation of numerous benign and malignant tumors, is characterized by loss of LKB1 kinase activity, a known upstream kinase and activator of AMPK^24^. However, LKB1 is not the only kinase that phosphorylates AMPK, and LKB1 phosphorylates numerous additional downstream targets that may contribute to its tumor suppressive role.

In contrast, AMPK activation was seen in early stages of glioblastoma tumor formation^25^, and AMPK activation was found to be critical for pancreatic cancer cell growth in anchorage-independent conditions^26^. Moreover, both AMPKα1^−/−^ and AMPKα2^−/−^ MEFs are resistant to Ras-induced oncogenic transformation, arguing that Ras-driven transformation requires AMPK^15,18^. Based on the conflicting evidence, AMPK has been described as a “conditional tumor suppressor and contextual oncogene^19^”. The cause of these conflicting reports may be due to the role of AMPK in stress response. In non-transformed cells, AMPK likely contributes to the maintenance of a non-transformed phenotype by promoting a controlled stress response. However, in transformed cells the stress response function of AMPK may promote survival in a suboptimal environment. While AMPKγ1 is required for colon cancer cell survival^6^, the contribution of other subunit isoforms on cancer cell survival has not been examined. We examined the expression and function of the AMPKα2 subunit in colon cancer cells and used FUSION to detect a competitive inhibitor of AMPK within a natural product library. This study highlights the potential of evaluating and targeting specific AMPK isoforms and serves as a proof-of-concept for FUSION-based detection of novel small molecule inhibitors of therapeutic targets.

## Results And Discussion

### AMPKα2 is differentially expressed, yet is required for survival in colon cancer cell lines

**Figure 1:**
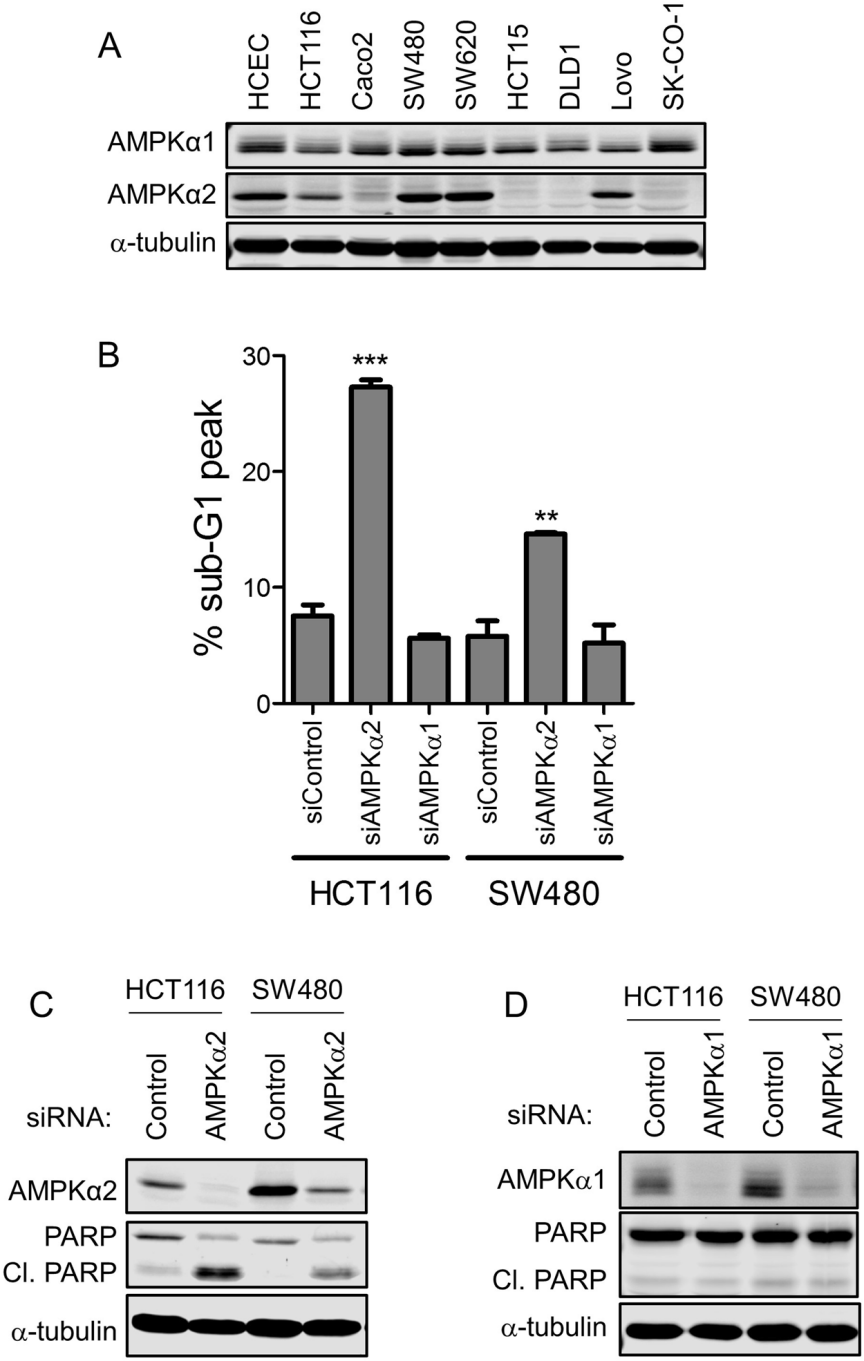
Differential effect of AMPK subunit depletion on colon cancer cell line survival. (**A**) AMPK subunit expression in a panel of colon cancer cell lines and HCECs. (**B**) Apoptosis (percent of cells in the sub-G1 peak) in HCT116 and SW480 cells after AMPKα1 or AMPKα2 depletion by RNAi for 72 hours. Apoptosis was evaluated using propidium iodide staining followed by flow cytometry analysis. (**C**) Immunoblot of AMPKα2 expression and PARP cleavage in HCT116 and SW480 cells following RNAi-mediated AMPKα2 depletion for 72 hours. (**D**) Immunoblot of AMPKα1 expression and PARP cleavage in HCT116 and SW480 cells following RNAi-mediated AMPKα1 depletion for 72 hours.

AMPK functions as a heterotrimeric complex consisting of a catalytic α subunit that possesses kinase activity and regulatory β and γ subunits^9^. The α2 AMPK subunit, but not the α1 subunit, promotes the survival of HCT116 colon cancer cells^6^. To further examine the importance of the individual AMPK alpha subunits, we examined the expression of AMPKα1 and AMPKα2 subunits in a panel of colon cancer cell lines. AMPKα1 expression was relatively consistent across cancer cells lines and was comparable to immortalized, non-transformed human colon epithelial cells (HCECs) expression (Fig. 1A). However, the expression of AMPKα2 was variable between cancer cell lines. The highest expression was observed in the SW480 and SW620 cancer cells. Moderate expression was demonstrated in non-transformed HCECs, as well as the Lovo and HCT116 cancer cells. Caco2, HCT15, DLD1 and SK-CO-1 cells demonstrated low AMPKα2 expression (Fig. 1A). Cell lines with moderate (HCT116) and high (SW480) AMPKα2 expression were selected to evaluate the effect of AMPK depletion or AMPK inhibition on colon tumor cell viability.

Propidium iodide staining followed by flow cytometry analysis and PARP cleavage were used to evaluate cell death after AMPKα1 or AMPKα2 depletion by RNAi for 72 hours. Regardless of the level of AMPKα2 expression, AMPKα2 depletion increased cell death in both colon cancer cell lines, while AMPKα1 depletion did not (Fig. 1B and C). These data indicate that AMPKα2, and therefore AMPK activity, is required for colon cancer cell survival even though individual AMPK subunits (AMPKα1) may be dispensable. Thus, identifying a compound that selectively inhibits the functional AMPK heterotrimer may be an efficacious therapeutic strategy to selectively target cancer cells.

### FUSION identifies a natural product that inhibits AMPK kinase activity

Functional Signature Ontology (FUSION) detects functional relationships between genes and microRNAs based on changes to a gene expression-based functional signature^6,7,27^. Previously, FUSION identified AMPKγ1 as a genetic functional analog of KSR1 based on unsupervised hierarchical clustering and quantification of similarity metrics (Euclidean distance and Pearson correlation) based on reporter gene expression following RNAi-mediated depletion of individual genes from a genome-scale human siRNA library. Biological validation demonstrated AMPKγ1 is also required for the survival of colon tumor cells, but not immortalized, non-transformed colon epithelial cells^6^. We hypothesized this approach could be used to identify small molecule inhibitors that mimic the effects of AMPK inhibition and are preferentially toxic to human colon tumor cells. As a proof-of-concept experiment, reporter gene expression signatures were generated for 1,186 unique chemical fractions isolated from a natural product library derived from a diverse selection of marine bacteria (*c*.*f*., Supplementary Methods)^7^. Comparing the gene expression signature of Compound C (also known as Dorsomorphin), a drug known to inhibit AMPK^14^, with fractions isolated from the natural product library, FUSION identified several fractions whose biologic activity was similar to Compound C treatment^7,28^.

**Figure 2:**
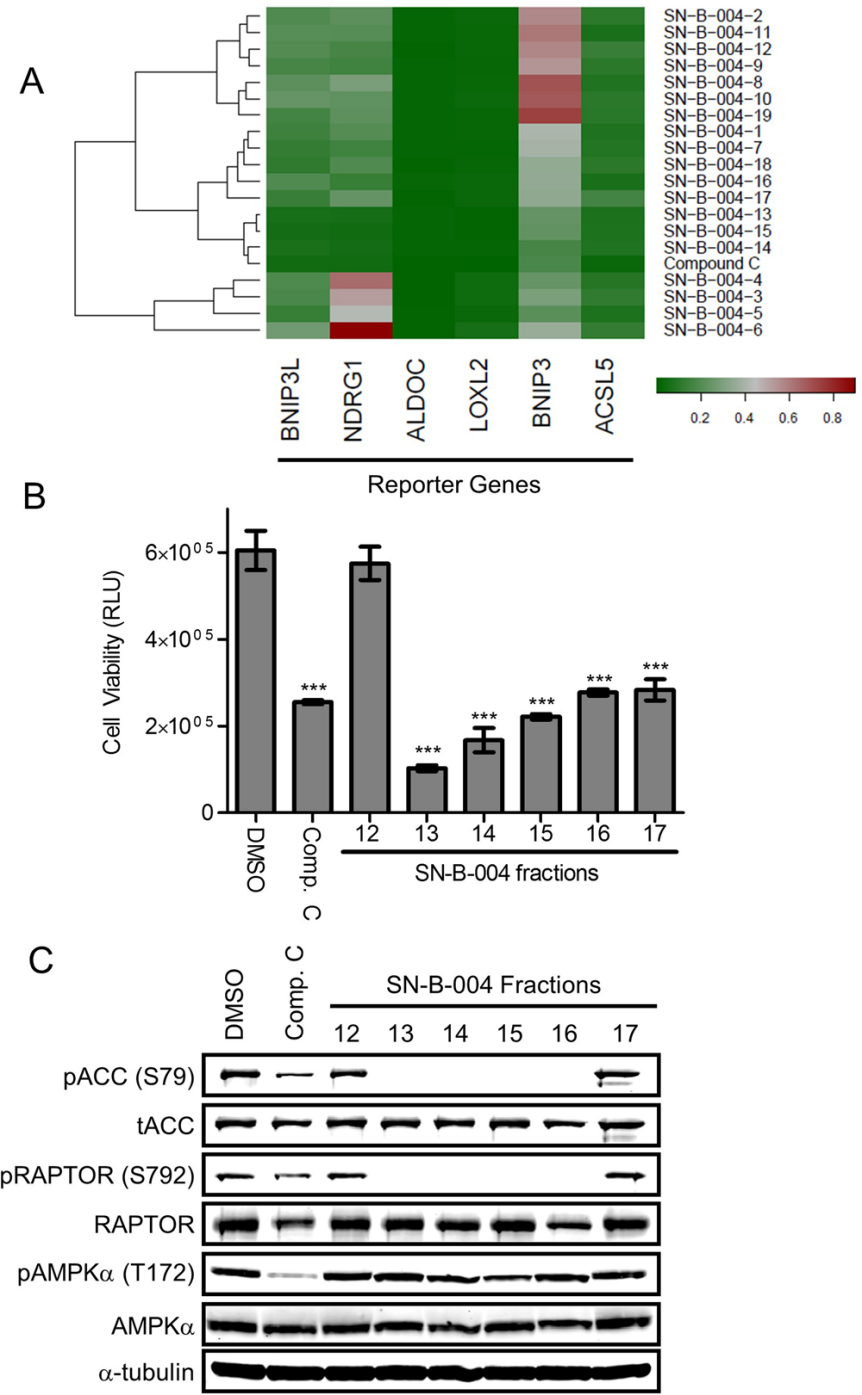
FUSION identifies an AMPK inhibitor. (**A**) Unsupervised hierarchical clustering of fractions isolated from the *Streptomyces bacillaris* strain SN-B-004 with Compound C. (**B**) Cell viability assay in HCT116 cells treated for 24 h with 10 μM of the indicated natural product fractions. Data are shown as mean relative light units (RLU) ± SD. ***p < 0.001. (**C**) Immunoblots of total and phosphorylated ACC (Ser79), RAPTOR (Ser792) and AMPK (Thr172) in HCT116 cells treated for 48 h with 10 μM of the indicated natural product fractions.

Several fractions isolated from the *Streptomyces bacillaris* strain SN-B-004 clustered with Compound C (Fig. 2A). Based on this observation, we hypothesized that the SN-B-004 fractions that clustered with Compound C contained an inhibitor of AMPK. Treatment with SN-B-004 fractions 13–17 decreased viability of colon tumor cell line HCT116 (Fig. 2B) and SN-B-004 fractions 13–16 decreased phosphorylation of two AMPK substrates acetyl-CoA carboxylase (ACC) at Ser79 and RAPTOR at Ser792 (Fig. 2C). However, these fractions appeared pharmacologically and mechanistically distinct from Compound C because they demonstrate a limited ability to prevent the phosphorylation of AMPKα at Thr172 (Fig. 2C), which is critical for AMPK activity^29^, while Compound C was able to decrease phosphorylation at Thr172. While Compound C has been shown to inhibit AMPK activity by decreasing phosphorylation on downstream targets, the mechanism behind its inhibition has not been fully elucidated. Increasing activating AMPK signals (AICAR or metformin treatment) is sufficient to overcome inhibition of Compound C suggesting that it does not directly inhibit the kinase activity of AMPK, but instead may act by regulating the activation of AMPK itself, which is likely based on its detrimental effect on AMPK phosphorylation^14^. In contrast, direct inhibition of the kinase activity of AMPK would lead to decreased phosphorylation of downstream targets of AMPK without directly affecting AMPK phosphorylation, though it may paradoxically increase the phosphorylation of AMPK itself due to loss of negative feedback loops.

**Figure 3:**
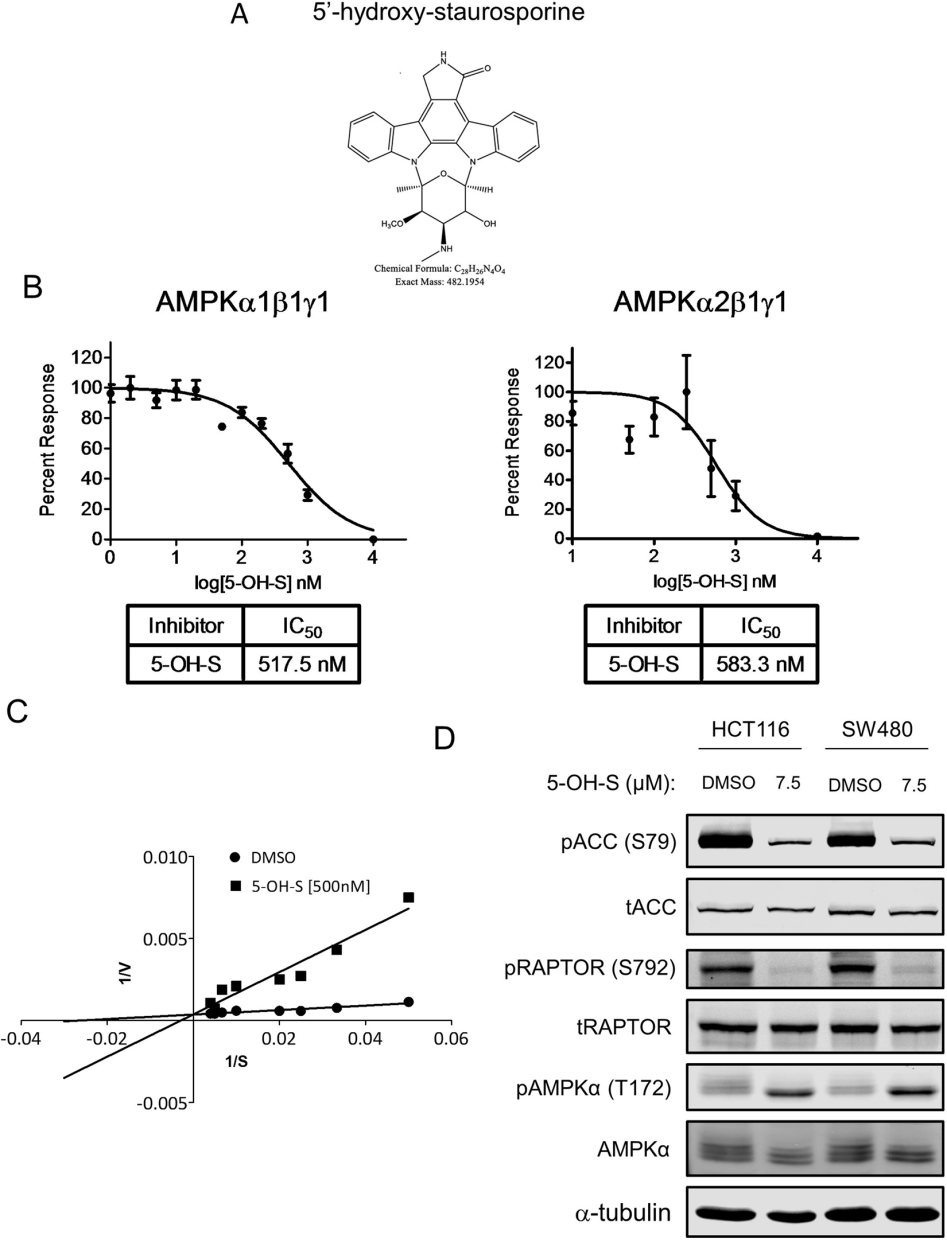
The identified active molecule, 5-OH-S, inhibits AMPK kinase activity. (**A**) Structure of 5-OH-S. (**B**) Dose-dependent inhibition of recombinant AMPKα1β1γ1 and recombinant AMPKα2β1γ1 kinase activity by 5-OH-S. (**C**) Lineweaver-Burke plots of AMPK substrate phosphorylation in the presence of DMSO or 500 nM 5-OH-S. (**D**) Immunoblots of total and phosphorylated ACC (Ser79), RAPTOR (Ser792) and AMPK (Thr172) after 48-hour treatment with 7.5 μM 5-OH-S in HCT116 and SW480 cells.

The active compound within the impure fraction SN-B-004-16 was isolated and the structure was determined using mass spectrometry and nuclear magnetic resonance spectroscopy to be 5′-hydroxy-staurosporine^30^ (5-OH-S, Fig. 3A and Supplemental Fig. 3), a derivative of the well-known, non-specific kinase inhibitor staurosporine^31^. To date, 5-OH-S has only been described in one other report in the literature in which it was isolated from another marine bacteria *Micromonospora* sp. strain L-31-CLCO-002^30^.

To determine if 5-OH-S directly inhibits AMPK kinase activity, we performed *in vitro* kinase assays of AMPK using SAMS peptide as a substrate, in the presence or absence of 5-OH-S. The IC_50_ of 5-OH-S for recombinant AMPKα1β1γ1 and AMPKα2β1γ1 were similar at 517.5 nM and 583.3 nM, respectively (Fig. 3B). The K_i_ for 5-OH-S inhibition of ATP binding to recombinant AMPK α1β1γ1 was 347 nM (Fig. 3C).

Similar to the SN-B-004 fractions, 5-OH-S decreased the phosphorylation of ACC at Ser79 and of RAPTOR at Ser792, known AMPK downstream targets, without decreasing the phosphorylation of AMPK at Thr172 in colon cancer cell lines HCT116 and SW480 (Fig. 3D).

### AMPK inhibition via 5-OH-S treatment is selectively toxic to colon cancer cells

**Figure 4:**
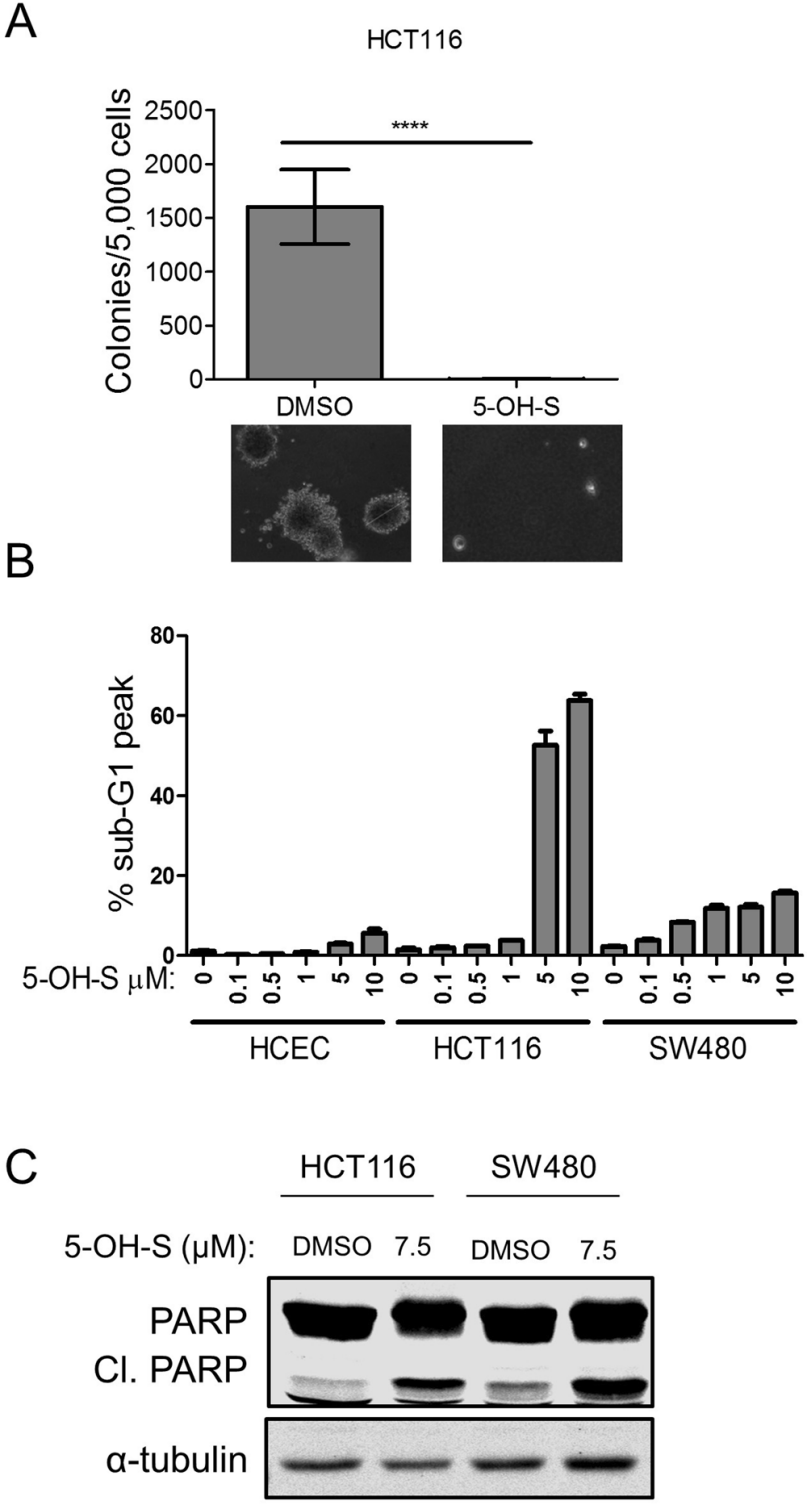
5-OH-S treatment preferentially inhibits colon cancer cell survival. (**A**) Colony formation following treatment with 7.5 μM 5-OH-S. ****p < 0.0001. (**B**) Dose-dependent apoptosis in HCT116 and SW480 colon cancer cells and HCECs following treatment with 5-OH-S. (**C**)Immunoblot of PARP cleavage in HCT116 and SW480 cells following treatment with 7.5 μM 5-OH-S for 48 hours.

AMPKγ1 is selectively required for colon cancer cell survival, but not HCECs survival^6^. We predicted that tumor cells would also be selectively sensitive to 5-OH-S as an inhibitor of AMPK. Treatment with 5-OH-S inhibited anchorage independent growth of HCT116 cells in a soft agar assay (Fig. 4A). Treatment with 5-OH-S decreased AMPK kinase activity in all cell lines tested as illustrated by reduced phosphorylation of ACC (Fig. 3D). However, 5-OH-S was preferentially toxic to the colon cancer cells lines (HCT116 and SW480) as compared to the HCECs (Fig. 4B). The induction of cell death following 5-OH-S treatment was verified in HCT116 and SW480 cells by analyzing PARP cleavage (Fig. 4C), which demonstrated increased PARP cleavage with 5-OH-S treatment.

These data suggest that either AMPK plays a larger role, or has additional mechanisms of action, that are required for cell survival in colon cancer cells but not in non-transformed colon epithelial cells. It is possible that some colon cancer cells have acquired oncogenic addiction to AMPK or one or more of the pathways modulated by AMPK. Importantly, these data demonstrate that FUSION has the potential to identify small molecules that are selectively toxic to tumor cells compared to non-transformed cells of the same lineage. The success of this proof-of-concept screen in identifying 5-OH-S from a limited natural product library suggests that the same approach, applied to a larger library, may yield novel agents with potent and selective therapeutic potential.

## Conclusions

Our data show that AMPK promotes the survival of multiple human colon cancer cell lines and that variable levels of AMPK alpha subunits may contribute to or predict their relative sensitivity to AMPK depletion. Tumor cells that have increased or altered AMPK subunit expression, or have employed alternative mechanisms to circumvent AMPK-regulated pathways, may overcome or lose sensitivity to AMPK depletion or inhibition. This suggests a role for AMPK isoforms with specific subunit composition and expression level in determining the contribution of AMPK toward tumor cell viability. This study provides additional evidence that cancer cells evolve diverse mechanisms to overcome obstacles limiting survival and proliferation. Cancer cells develop defined dependencies and vulnerabilities that offer a basis for their characterization and specific therapeutic intervention. In this study, we showed that colon cancer cells have variable requirements for the AMPK α2 subunit isoform for survival. This may reflect differing responses to environmental stresses that directed the tumor’s evolution and suggests that at least a subset of colon tumors may be highly susceptible to AMPK inhibition. This phenomenon is not likely to be limited to colon cancer as several other groups have demonstrated a requirement for AMPK or its downstream effects in both prostate and breast cancer and have shown that inhibition of AMPK is detrimental to cancer cells^32–36^.

In this study, we describe a novel, direct kinase AMPK inhibitor, 5′-hydroxy-staurosporine (5-OH-S), that has been isolated only from marine bacteria and has yet to be synthesized or made commercially available^30^. The effects of 5-OH-S appear to exceed those seen with individual AMPK subunit depletion. This is not surprising as 5-OH-S treatment inhibits both AMPK isoforms containing either α subunit and likely all AMPK trimer complexes. However, a detailed kinase inhibitor profiling to assess the effects of 5-OH-S on other targets has not been completed. Therefore, the possibility exists that 5-OH-S may have off-target effects that contribute to its anticancer effects. A structurally similar compound, 7′-hydroxy-staurosporine (also known as UCN-01^37^), has also previously demonstrated significant anti-cancer effects. Like other staurosporine derivatives, 7′-hydroxy-staurosporine has broad intracellular effects and inhibits multiple kinases, notably Protein Kinase C^38,39^. Regardless, 7′-hydroxy-staurosporine has been examined in numerous phase I and phase II trials for multiple types of cancer including T-cell lymphomas, leukemia, breast cancer, small cell lung cancer, melanoma, pancreatic cancer, kidney cancer, ovarian/fallopian tube cancer, and many other solid tumors^40–52^; however, its use has been constrained due to limited single agent efficacy, in conjunction with a less than optimal pharmacokinetic profile, and undesirable side effects. Our data suggest that staurosporine derivatives can act as lead compounds for the development of more specific AMPK kinase domain inhibitors with the goal of improved target specificity, anti-cancer efficacy, and reduced treatment complications.

The current study expands upon previous work that used FUSION to identify microRNAs and individual genes as potential therapeutic targets in cancer^6,7,27^. This study demonstrates the ability of FUSION to identify novel small molecules from an unbiased screen of crude natural product fractions that inhibit a specific target important for cancer cell survival. In this instance, FUSION identified 5-OH-S as an inhibitor of AMPK, which can serve as a lead compound that can be used to understand AMPK activity and could be further developed using medicinal chemistry for use as a cancer therapeutic. This proof-of-concept study suggests that applying FUSION analysis to a larger, more diverse library of small molecules and/or crude natural product fractions could identify numerous lead compounds that are more specific inhibitors of AMPK or other therapeutic targets, leading to the development of efficacious targeted therapies.

## Materials and Methods

### Cell culture and Reagents

HCT116, DLD1, SW480, SW620, SK-Co-1, Lovo, Caco2 and HCT15 cells were purchased from American Type Culture Collection (ATCC). HCECs were a generous gift provided by Dr. Jerry Shay (UT Southwestern Med. Ctr.). Cells were grown in either Dulbecco’s Modified Eagle’s Medium (DMEM), Eagle’s Minimum Essential Medium, or F12-K Medium with 10% fetal bovine serum (FBS), 2mM L-Glutamine, and 0.1 mM nonessential amino acids (NEAA). All colorectal cancer cells were grown at 37 °C with ambient O_2_ and 5% CO_2_. HCECs were cultured in 37 °C incubator with 2% oxygen and 5% CO_2_ in Basal X medium containing four parts of DMEM and one part of Medium 199 (#11150-059, Invitrogen, Carlsbad, CA) supplemented with 10% FBS, 2% Cosmic Calf Serum (#SH 30087, Hyclone, Logan UT), 25 ng/ml of recombinant EGF (#236-EG, R&D, Minneapolis, MN), 1 µg/ml hydrocortisone (#H0888), 10 μg/ml insulin (#I550), 2 μg/ml of Apo-Transferrin (#T1428), 5 nM sodium selenite (#S5261) all from Sigma.

### Antibodies and Reagents

Primary antibodies were used in the indicated dilutions: PARP (#9542, Cell Signaling) 1:1000, α-Tubulin (#B-5-1-2, Santa Cruz) 1:2500, pACC (#3661, Cell Signaling) 1:2000, tACC (#3676, Cell Signaling) 1:2000, pRAPTOR (#2083, Cell Signaling) 1:1000, tRAPTOR (#2280, Cell Signaling) 1:1000, pAMPKα1α2 (#2531, Cell Signaling) 1:1000, AMPKα1α2 (#2532, Cell Signaling) 1:1000, AMPKα2 (#AF2850, R&D systems) 1:1000, and AMPKα1 (#2795, Cell Signaling) 1:1000. Anti-mouse, and anti-rabbit secondary antibodies conjugated to Alexa Fluor 680 (Invitrogen, Carlsbad, CA) or IRDye800, and IRDye680LT were used at 1:5000-1:10,000 dilutions. Additional reagents included recombinant AMPKα1β1γ1 (#P47-10H, SignalChem) and AMPKα2β1γ1 (#P48-10H, SignalChem), SAMS peptide (S07-58, Cquential Solutions), and radioactive α-^32^P-ATP (64014, MPBIo).

### Immunoblot

Whole-cell lysate extracts were prepared in radioimmunoprecipitation assay (RIPA) buffer composed of 50 mM Tris-HCl, 1% NP-40, 0.5% Sodium deoxycholate, 0.1%

Sodium dodecyl sulfate, 150 mM NaCl, 2 mM EDTA, 50 mM NaF, 10 mM NaPPi, and 0.5 mM Na_3_VO_4_, 10 μg/mL aprotinin, 10 μg/mL leupeptin, 2 mM EDTA, 1 mM PMSF. Protein concentration was determined using the BCA protein assay (Promega). Samples were diluted in 1× sample buffer (5× stock = 250 mM Tris-HCl pH 6.8,10% SDS, 50% glycerol, 0.05% bromophenol blue) with 100 mM DTT (10× stock = 1 M). SDS-PAGE was performed and proteins were transferred to PVDF or nitrocellulose membranes. Membranes were blocked for greater than 45 minutes in Odyssey PBS blocking buffer (LI-COR Biosciences, 927-40000), and incubated in primary antibody (listed above) overnight at 4 °C. Secondary antibodies (listed above) were diluted 1:5000–1:10,000 in 0.1% TBS-Tween 20 (for nitrocellulose) or 0.1% TBST + 0.01% SDS (for PVDF). Membranes were imaged using the LI-COR Odyssey.

### siRNA transfection

Pooled or individual siRNAs targeting AMPKα1 (005027), AMPKα2 (005361), or a non-targeting siRNA control (001810-01) (DharmaconGE), were introduced into HCT116 or SW480 cells using the Lipofectamine RNAiMAX (Invitrogen) reverse transfection protocol per manufacturer’s instructions. Briefly, 125 pmol of siRNA and 5 μL of RNAiMax were combined in OPTI-MEM for 5 minutes. DNA:Lipofectamine complexes were overlaid with 2 mL of cells (150,000 cells/mL) in 6-well plates. Final RNAi concentrations are 50 nM. After a 72-hour transfection, cells were lysed in RIPA lysis buffer (described above) (Fig. 1B–D).

### Cell viability assay

Cells were plated on clear bottom, black-walled, 96-well plates. Natural product fractions were added 24 hours after plating and 48 hours later cell viability was measured. Cell viability was measured per the manufacturer’s protocol using the CellTiter-Glo® Luminescent Cell Viability Assay (Promega) by adding 90 µl of CellTiter-Glo® reagent, shaking for two minutes to lyse the cells, incubating at room temperature for 10 minutes, and measuring luminescence (POLARstar OPTIMA) (Fig. 2B).

### *In vitro* kinase assay

AMPK assays were performed by diluting 20 ng of AMPKα1β1γ1and 60 ng of AMPKα2β1γ1 in 5 µl of 10 mM MOPS (pH 7.2), 5 mM β-glycerophosphate, 10 mM MgCl_2_, 2 mM EGTA, 0.8 mM EDTA and 0.1 mM DTT, 80 ng/µl BSA and 8% glycerol and placing them on ice. 5 µl of AMP (480 µM final in water), drug or DMSO diluted in water (1:10,000 nM final), 1 mg/ml SAMS substrate (in water) and α-^32^P-ATP (40–500 µM final dilution in 25 mM MOPS pH 7.2, 12.5 mM β-glycerophosphate, 25 mM MgCl_2,_ 5 mM EDTA, 2 mM EGTA and 0.25 mM DTT) was added. Standard assay included a 50 µM final ATP concentration. Samples were mixed and incubated in 30 °C water bath for 15 minutes with gentle rocking and then returned to ice. 20 µl of samples were spotted on P81 paper and allowed to dry. Papers were washed three times each with 200 ml 0.1% phosphoric acid, allowed to dry, placed in a vial with scintillation cocktail, and counted. One sample without enzyme was used to correct for non-specific binding to the P81, which was determined to be equal to using no SAMS peptide in a mock assay (Fig. 3B).

### Anchorage-independent growth in soft agar

Cells were seeded at 5 × 10^3^ cells/35 mm dish in 1 ml of top agarose consisting of Iscoves’s Dulbecco Modified Growth Medium (DMEM) mixed with 0.4% NuSieve GTG agarose, 4 mM L-Glut, 1% NEAA and 1% penicillin/streptomycin suspended over a bottom layer consisting of 2 ml of DMEM with 0.8% Nu-Sieve GTG agarose, 4 mM L-Glut, 1% NEAA and 1% penicillin/streptomycin. DMSO or 5-OH-S was placed in both top and bottom layers at a concentration of 10 µM. Colonies over 100 microns were counted and representative photomicrographs were taken after 14 days of incubation in 37 °C and 5% CO_2_ (Fig. 4A).

### Cell cycle analysis using propidium iodide staining

Cells were treated as indicated for 72 hours before being harvested. Cells in suspension were collected with the trypsin-treated adherent cells, washed with 1× PBS, and then fixed with 70% ice-cold ethanol at −20 °C for 1 hour to overnight. Cells were then pelleted and rehydrated with 1× PBS at 37 °C for 15 minutes. Cells were again pelleted and mixed with TELFORD reagent (1% Triton-X-100, 33.6 mg/l EDTA, 26.8 mg/l RNAse A and 50 mg/ml propidium iodide in 1× PBS) and stored overnight at 4 °C before analysis by flow cytometry (Figs 1B and 4B).

### Statistics

The CellTiter-Glo® Luminescent Cell Viability Assay (Promega) and apoptosis was statistically evaluated using a one-way ANOVA with Dunnett’s post-test (Figs 1B and 2B). Significance of colony formation on soft agar was calculated using unpaired, two-sided t-test (Fig. 4A).

### Data availability

The datasets generated during and/or analyzed during the current study are available from the corresponding author on reasonable request.

## Electronic supplementary material

Supplementary Materials
