# Supplementary material for: A Functional Signature Ontology (FUSION) screen detects an AMPK inhibitor with selective toxicity toward human colon tumor cells
Source: Sci Rep. 2018 Feb 28;8:3770. doi: 10.1038/s41598-018-22090-6 (PMC5830883; doi:10.1038/s41598-018-22090-6)
Supplement: Supplementary file 1 — Supplementary Materials [file 41598_2018_22090_MOESM1_ESM.pdf]

A Functional Signature Ontology (FUSION) screen detects an AMPK inhibitor with selective toxicity toward human colon tumor cells

Binita Das[1,a,†], Beth K. Neilsen[1,†], Kurt W. Fisher[1,c], Drew Gehring[1], Youcai Hu[2,b], Deanna J. Volle[1], Hyun Seok Kim[3,d], Jamie L. McCall[1,e], David L. Kelly[1], John B. MacMillan[2], Michael A. White[3], and Robert E. Lewis[1,*]

SUPPLEMENTARY MATERIALS

Materials and Methods: Natural Product Screen and 5´-hydroxy-staurosporine isolation
Table S1. Sequences of siRNA oligonucleotides
Supplementary Figures 1-4

Natural products library
The natural products fraction library used for this study is composed of extracts from 92 marine-derived bacterial strains and 20 marine invertebrates (19 sponges, 1 tunicate). The library of microbial NP fractions was derived from marine-derived Actinomycetes (65), Firmicutes (20) and alpha-proteobacteria (7). These bacteria were cultivated from marine sediment samples collected in the Gulf of Mexico (Texas, Louisiana), estuaries in South Carolina and the Bahamas. Small-molecule signaling compounds (*N*-acylhomoserine lactones, siderophores, and surfactins) were used to mimic the natural environment of the bacteria of interest. Use of nutrient-limited isolation media, such as those composed of only humic or fulvic acid was used for isolation of actinomycetes and alpha-proteobacteria from mangrove and estuary sediments. The majority of bacterial isolates were characterized using 16S rRNA phylogentic analysis following previously described methods (*64*). Universal 16S rRNA primers FC27 and RC 1492 were used. 16S rRNA sequences were compared to sequences in available databases using the Basic Local Alignment Search Tool. Specific details of the strain isolation and phylogenetics for bacteria discussed in the text are included below. For bacterial isolates not characterized by 16S rRNA, we have used morphology to ensure that strains in the collection are not duplicated.

To generate the fraction library, bacterial strains were fermented in 5 × 2.8 L Fernbach flasks each containing 1 L of a seawater based medium (10 g starch, 4 g yeast extract, 2 g peptone, 1 g $CaCO_3$, 40 mg $Fe_2(SO_4)_3{\cdot}4H_2O$, 100 mg KBr) and shaken at 200 rpm for seven days at 27 ºC. After seven days of cultivation, XAD7-HP resin (20 g/L) was added to adsorb the organic products, and the culture and resin were shaken at 200 rpm for 2 h. The resin was filtered through cheesecloth, washed with deionized water, and eluted with acetone to give a crude extract. Further fractionation of the bacterial crude extracts was accomplished using an Isco medium pressure automatic purification system using reversed phase C_{18} chromatography (gradient from 90:10 H_2O:CH_3CN to 0:100 H_2O:CH_3CN over 25 minutes). Fermentation of each bacterial strain gives rise to a total of 20 natural product fractions/strain for further testing. All natural product fractions in the library are standardized to a concentration of 10 mg/mL in DMSO.

Producing organism
Standard procedures for 16S rRNA analysis were used for phylogenetic characterization of bacterial strains(*65*). Bacterial strain SNB-004 was derived from a sediment sample collected in Galveston Bay, TX in 2008. Based on 16S rRNA analysis the strain was determined to be *Salinispora arenicola.*

Purification of 5´-hydroxy-staurosporine from SNB-004

Strain SNB-004 was isolated from a sediment sample collected from a sediment samples collected in Galveston Bay, TX. A desiccated sediment sample was stamped on a seawater based media (10 g starch, 4 g yeast extract, 2 g peptone, 15 g agar, 1 L sH_2O) containing rifampicin (50 mg/L) and cycloheximide (10 mg/L). Phylogentic analysis using 16S rRNA analysis revealed the strain to be identical to *S. arenicola*. The dried crude extract from 10 L of *S. arenicola* (5.8 g) was purified using solvent partition (MeOH), the methanol soluble portion (2.5 g) was further partitioned using EtOAc and H_2O (1:1 mixture). The ethyl acetate layer (0.9 g) was purified via reversed phase flash column chromatography, eluting with a step gradient of H_2O and MeOH (90:10-100:0) collecting 9 fractions. Fraction 4 (SNB-004-4, 72.4 mg) was purified by reversed phase HPLC (Phenomenex Luna, C18, 250×21.2 mm, 8.0 ml/min, 10µm, UV=254nm) using a gradient solvent system from 25% to 60% MeOH (0.1% formic acid) over 28 min, collecting 7 fractions, of which the active fraction was 5´-hydroxy-staurosporine.

5´-hydroxy-staurosporine: λ_{max} (log ε) 220 (6.2), 240 (2.3), 300 (2.7), 322 (2.6). ESI-MS *m/z* 483.2 $[M + H]^+$, 481.2 $[M – H]^-$. HRESIMS *m/z* 483.5485 $[M + H]^+$. ^1H NMR was identical to literature reports of 5´-hydroxy-staurosporine (Hernandez, *The Journal of Antibiotics*, 2000) and an authentic standard.

TABLE S1: Sequences of siRNA oligonucleotides

Target	Item #	siRNA	Sequence
AMPKα1	J-005027	6	CCAUACCCUUGAUGAAUUA
		7	GCCCAGAGGUAGAUAUAUG
		8	GAGGAUCCAUCAUAUAGUU
		9	ACAAUUGGAUUAUGAAUGG
AMPKα2	J-005361	6	CGACUAGCCCAAAUCUUU
		7	GAGCAUGUACCUACGUUAU
		8	GACAGAAGAUUCGCAGUUU
		9	GUCUGGAGGUGAAUUAUUU

Supplementary Figures 1-4

Figure 1A

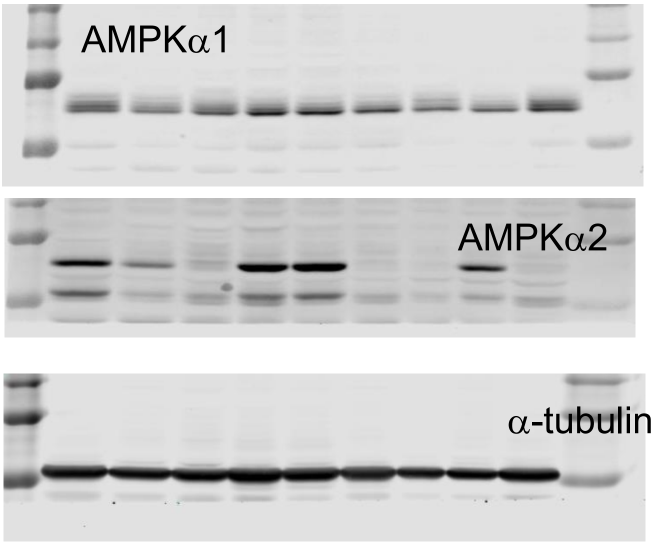

Figure 1C – First Four Lanes

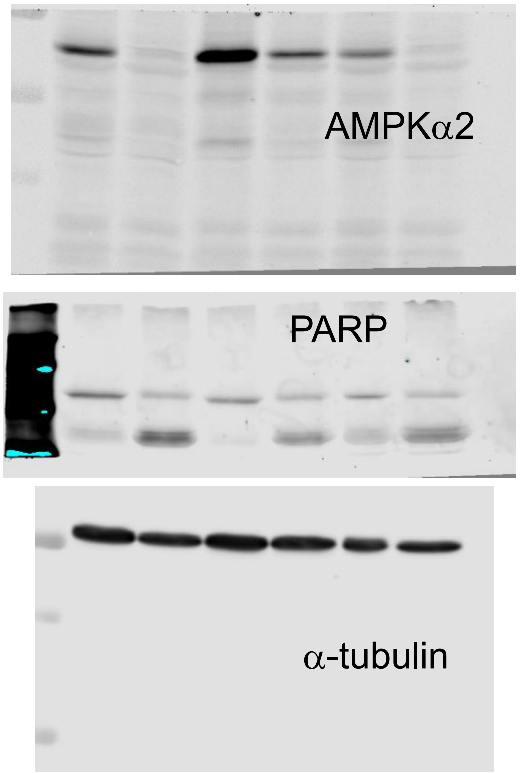

Figure 1D – First Four Lanes

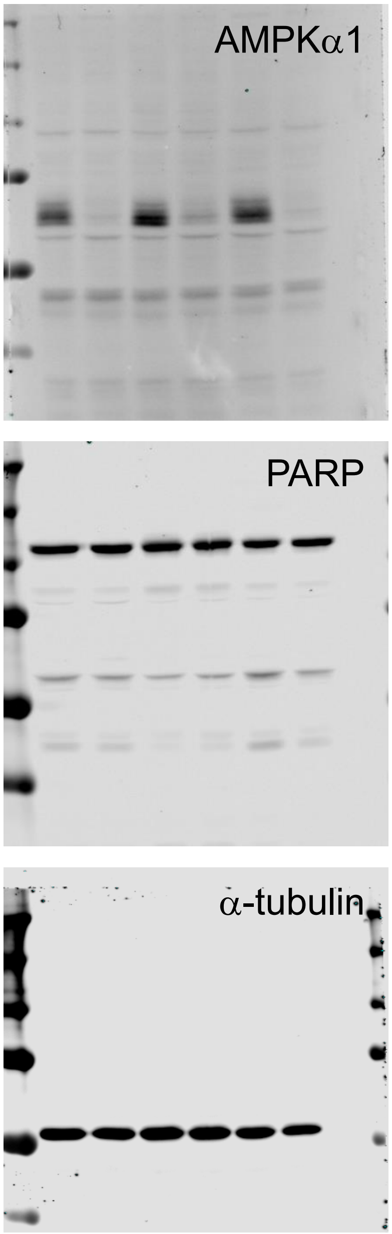

Supplementary Fig. 1. Whole immunoblots for Fig. 1.

Figure 2C

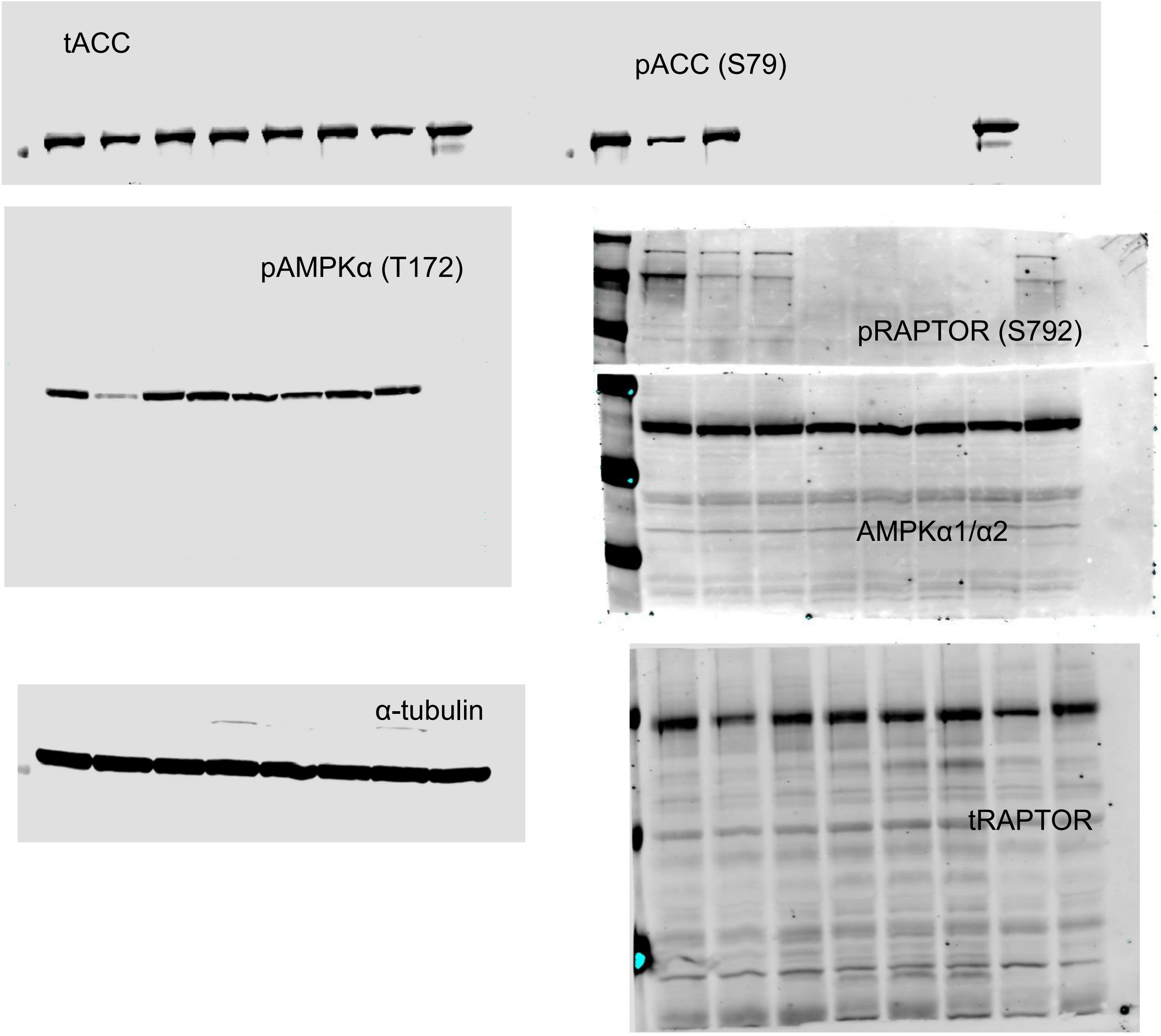

Supplementary Fig. 2. Whole immunoblots for Fig. 2.

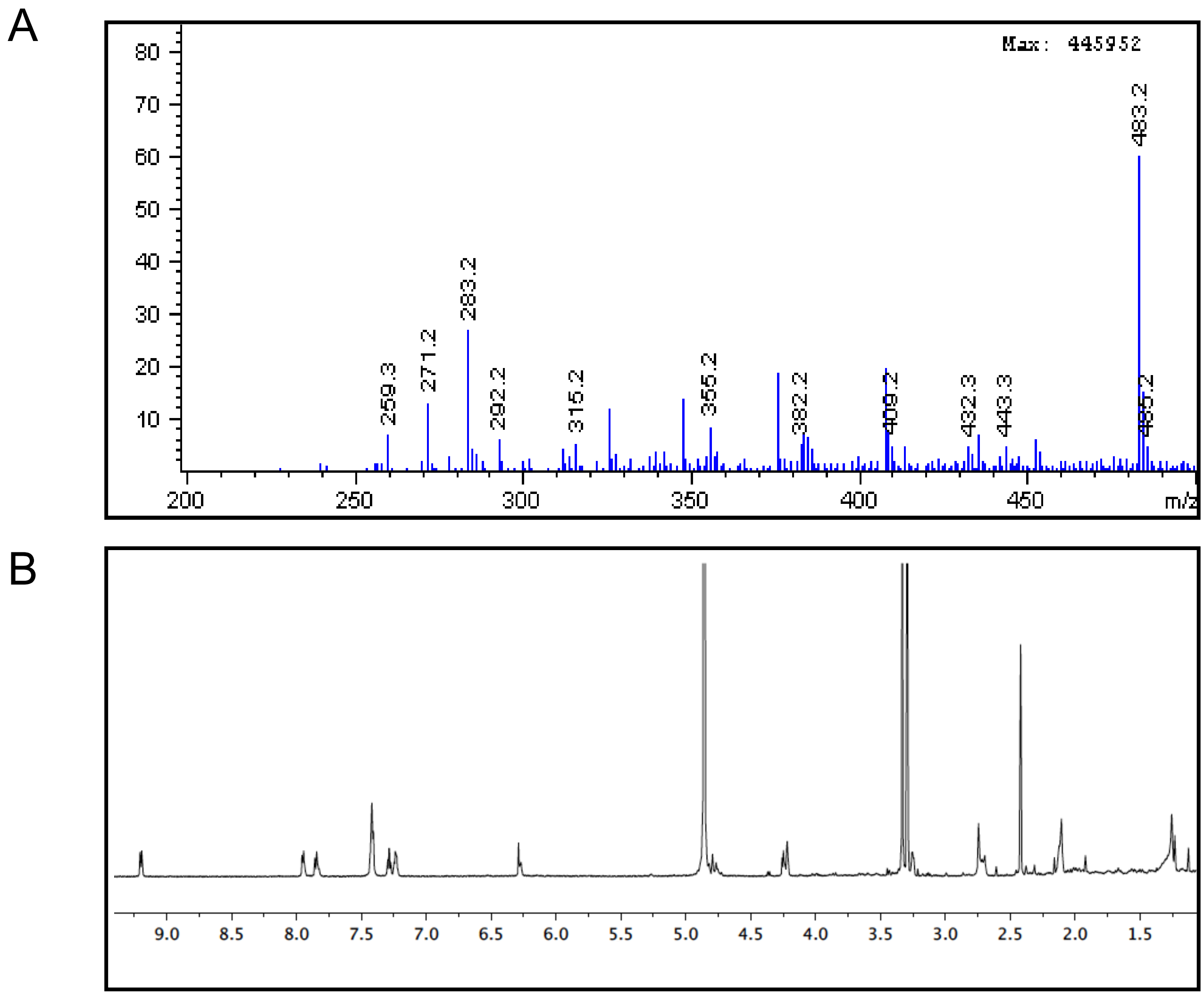

Supplementary Fig. 3. Identification of the active molecule in natural product library fraction SN-B-004-16 identified by FUSION. (A) ESI-MS $[M+H]^+$ of 5-OH-S. (B) ^1H NMR of 5-OH-S at 600 MHz in CD_3OD.

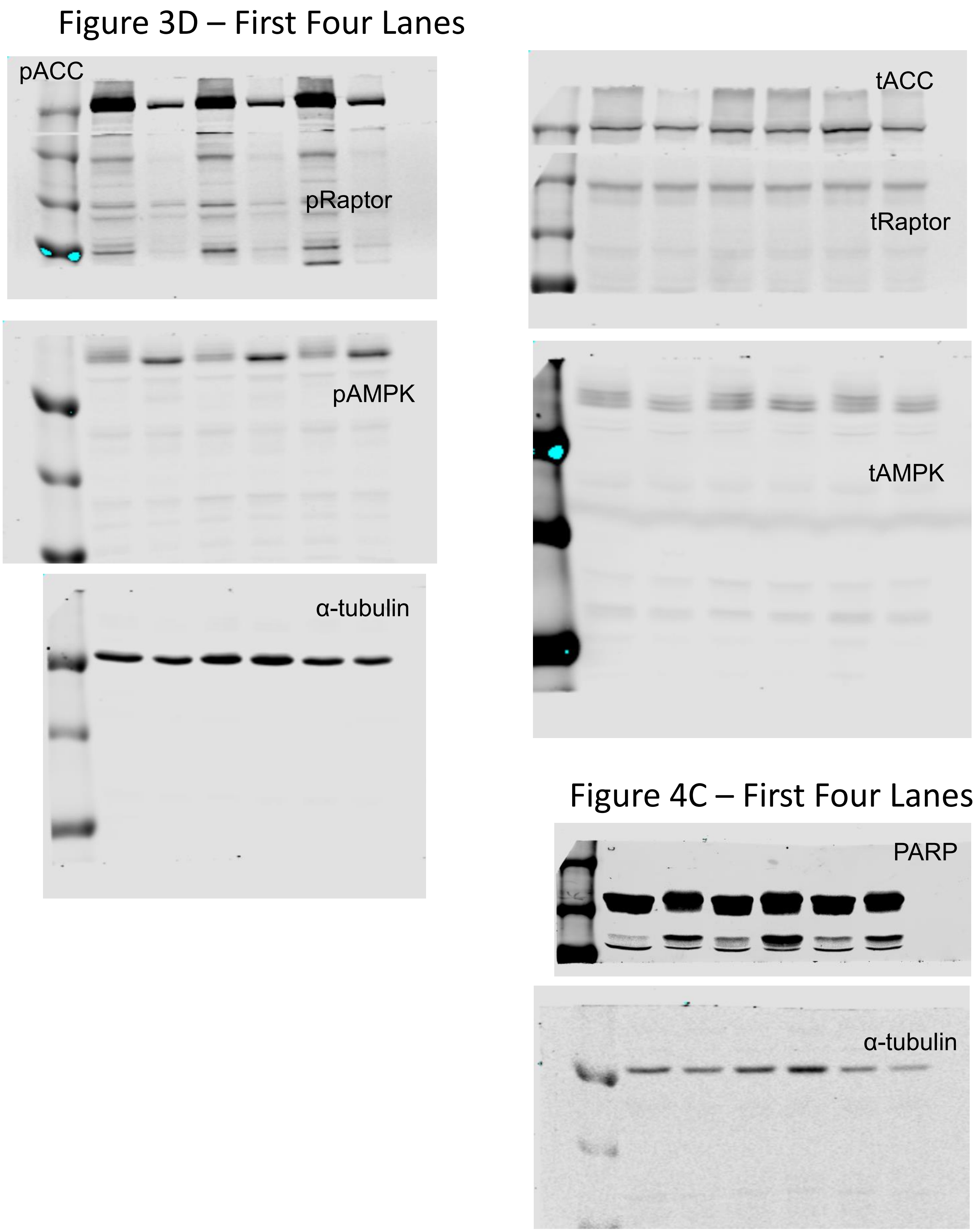

Supplementary Fig. 4. Whole immunoblots for Fig. 4.
